# Development of targeted therapy for ovarian cancer mediated by a plasmid expressing diphtheria toxin under the control of H19 regulatory sequences

**DOI:** 10.1186/1479-5876-7-69

**Published:** 2009-08-06

**Authors:** Aya Mizrahi, Abraham Czerniak, Tally Levy, Smadar Amiur, Jennifer Gallula, Imad Matouk, Rasha Abu-lail, Vladimir Sorin, Tatiana Birman, Nathan de Groot, Abraham Hochberg, Patricia Ohana

**Affiliations:** 1 The Department of Biological Chemistry, Institute of Life Sciences, Edmond Safra Campus, Givat Ram, Jerusalem 91904, Israel; 2 Sheba Medical Center, Department of General and Hepatobiliary Surgery, Tel Hashomer 52621, Israel; 3 E. Wolfson Medical Center, Genecology Oncology, Holon 58100, Israel; 4 E. Wolfson Medical Center, Department of Surgery "A", E. Wolfson Medical Center, Holon, Israel

## Abstract

**Background:**

Ovarian cancer ascites fluid (OCAF), contains malignant cells, is usually present in women with an advanced stage disease and currently has no effective therapy. Hence, we developed a new therapy strategy to target the expression of diphtheria toxin gene under the control of H19 regulatory sequences in ovarian tumor cells. H19 RNA is present at high levels in human cancer tissues (including ovarian cancer), while existing at a nearly undetectable level in the surrounding normal tissue.

**Methods:**

H19 gene expression was tested in cells from OCAF by the in-situ hybridization technique (ISH) using an H19 RNA probe. The therapeutic potential of the toxin vector DTA-H19 was tested in ovarian carcinoma cell lines and in a heterotopic animal model for ovarian cancer.

**Results:**

H19 RNA was detected in 90% of patients with OCAF as determined by ISH. Intratumoral injection of DTA-H19 into ectopically developed tumors caused 40% inhibition of tumor growth.

**Conclusion:**

These observations may be the first step towards a major breakthrough in the treatment of human OCAF, while the effect in solid tumors required further investigation. It should enable us to identify likely non-responders in advance, and to treat patients who are resistant to all known therapies, thereby avoiding treatment failure.

## Background

Epithelial ovarian cancer (EOC) is the second most common gynecologic cancer, with an estimated 22,000 new cases and 15,000 deaths per year in the United States [1]. The median age of patients with ovarian cancer is 60 years old, and the average lifetime risk for the development of EOC is about 1 in 70, with an overall five year survival rate not exceeding 35% [2].

The peritoneal cavity is a common site of ovarian cancer presentation or recurrence usually accompanied by ascites [3]. Massive ascites and the associated abdominal distention can cause anorexia, nausea, vomiting and respiratory difficulties, affecting the patient's quality of life [4]. EOC patients frequently have involvement of the pelvic and retroperitoneal lymph nodes as well [5,6]. The standard primary treatment of patients with advanced stage EOC is cytoreductive surgery followed by platinum and taxane doublet chemotherapy. Despite this aggressive approach, there is a high rate of recurrence. Although discovery of several other active nonplatinum cytotoxic agents has improved outcome [7], long-term survival rates are still disappointing and most women will die as a result of their disease. Success of traditional chemotherapy has been limited by drug resistance and lack of specificity to mechanisms of disease formation and progression. Thus, novel targeted therapies are extensively explored in order to achieve improved long-term control with lower toxicity.

An attractive approach to human cancer gene therapy is to exploit the genetic and epigenetic alterations in a cancer for targeting the expression of toxic genes. Indeed, several attempts have been made in this direction, employing e.g. promoters of the telomerase (hTERT) gene or promoters induced by hypoxia-inducible factors [7,8].

We developed a novel therapy approach based on patient-specific gene expression profiles in each cancer tailored to individual patients by using selected transcriptional regulatory sequences for DNA-based therapy. This enables the directing of a tumor-selective expression of a toxin, delivered by a non-viral vector. Non-viral vectors appear promising due to their potential to overcome the main disadvantage of adenoviral vectors, causing immune responses directed against adenovirus proteins, and limit their ability to be administered iteratively.

Based on earlier studies from our group and others, transcriptional regulatory sequences of the H19 gene have emerged as candidates for cancer gene therapy. H19 is a paternally-imprinted, maternally expressed, oncofetal gene that encodes a RNA acting as "riboregulator" that has no protein product [9]. It is expressed at substantial levels in several different human tumor types, but is only marginally or not at all expressed in normal adult tissues [10,11]. Its precise function has been debated. Recent data suggested a role for H19 in promoting cancer progression, angiogenesis and metastasis [12,13].

The human H19 gene lies within 200 kb downstream of the paternally expressed IGF2 gene at 11p.15.5. Shared enhancers downstream to H19 coordinate transcription of both genes [14]. The list of cancers in which H19 gene expression is known to be elevated compared to normal tissue is still growing [11,15-18]. Detection of H19 expression in epithelial ovarian cancer using ISH technique revealed that H19 is expressed in the majority of serous epithelial tumors [19].

As a toxic gene, we chose the diphtheria toxin A chain (DT-A), which has suitable properties for achieving efficacious cancer cell killing [20,21]. Thus, using a combination of therapeutic expression constructs driven by promoters differentially expressed and gene expression profiling allows an individualized DNA-base approach to cancer therapy. The therapeutic potential of the DTA-H19 vector was tested in a rat animal tumor model for colorectal liver metastases showing tumor growth inhibition in the DTA-H19 treated group as compared to the control group [22]. The safety, tolerability and preliminary efficacy of the therapuetic vector DTA-H19 was tested successfully in a phase 1/2a clinical trial for the treatment of superficial transitional cell carcinoma (TCC) of the bladder [23,24] and, based on these results, a multicenter phase 2b clinial study has been initiated.

The therapeutic potential of a vector carrying the DT-A gene driven by H19 regulatory sequences was tested both in ovarian cancer cell lines and in a subcutaneous nude mice model for ovarian cancer. The results showed high killing potential in ovarian cancer cell lines and a significant tumor growth inhibition in animals, indicating that the DTA-H19 construct has a high therapeutic potential and is a very promising candidate for ovarian cancer therapy in humans.

## Materials and methods

### Cell culture

The human ovarian carcinoma cell lines (ES-2, SKOV-3, TOV-112D and OVCAR-3) used in this study were obtained from the American type culture collection (ATCC). Cells were maintained in DMEM-F12 (1:1) medium containing 10% fetal calf serum. For OVCAR-3, 0.01 mg/ml of human insulin was added to the culture medium.

### Plasmids and constructs

All the luciferase gene reporter constructs were built from the pGL3 basic (Luc-1) vector (Promega, Madison, WI, USA) which lacks both promoter and enhancer sequences. The construct Luc-H19 contains the reporter gene under the control of the human H19 promoter region from nucleotide -818 to +14 was prepared as described [25]

The Luc-H19 plasmid was digested with Xba I and Nco I and the insert of the luciferase gene (*luc*) was replaced by the diphtheria toxin A-chain (*DT-A*) coding region to yield the DTA-H19 construct. Large-scale preparations of the plasmids were performed using the EndoFree Plasmid Mega kit (Quiagen, Germany). All plasmids were modified by replacing the Amp res gene by the Kan res gene.

### In vitro transfection and luciferase assay

A total of 1*10^5 cells were plated in a twelve-well Nunc multidish (30 mm). Transient transfections were carried out using the JetPEI cationic polymer transfection reagent (mean molecular weight of 22 kDa; Polyplus, Illkirsh, France). The transfection was carried out according to the manufacturer's instructions using 2 μg of DNA and 3 μl of JetPEI solution to obtain a N/P ratio of 5. Transfection experiments were stopped after 48 h and reporter gene activity was assessed. Luciferase activity was measured using the Promega kit 'Luciferase Assay System' (E-1500; Promega, Madison, USA). Light output was detected using a Lumac Biocounter apparatus. Protein content was measured by the Bio-Rad (Hercules, CA, USA) protein assay reagent, and the results were expressed as light units/μg protein. LucSV40 (Luc-4) was used as a reference for maximal luciferase activity, as it contains the SV40 promoter and enhancer, while Luc-1 lacking regulatory sequences was used as a negative control to determine the basal non-specific luciferase expression, which was found to be negligible. All experiments were carried out in triplicate and the results represent the mean value and standard error was calculated. In all the transfection experiments the measured Luciferase activity is expressed as a percentage of that observed after transfection with the positive control plasmid (Luc-4) alone, to allow normalization of luc activity.

### In vitro activity and specificity of the regulatory sequences (cell killing assay)

Cells were cotransfected using 2 μg of the reporter vector Luc-4 and the indicated amounts of the DTA-H19 expression vector using the transfection reagent JetPEI as described above. Cells were also transfected by 2 μg of Luc-4 alone. *In vitro *activity of the regulatory sequences was determined by calculating the % decrease in the luciferase activity in the cotransfected cells compared with that of the cells transfected only with Luc-4.

### RNA isolation and cDNA synthesis

Total RNA was extracted from cell lines or tissues, using the RNA STAT-60™ using total RNA/mRNA isolation reagent (Tel-Test, Inc., Friendswood, TX, USA), according to the manufacturer's instructions. The RNA was treated with RNase-free DNase I (Roche Diagnostics GmbH, Mannheim, Germany) to eliminate any contaminating DNA. The cDNA was synthesized from 2 μg total RNA in 20 μl reaction volume as described [24]

### Determination of the level of RNA products of the H19 gene

The PCR reactions were carried out in 25 μl volumes in the presence of 6 ng/μl of each of the forward and the reverse primers using 0.05 units/μl of Taq polymerase (TaKaRa Biomedicals, Japan) according to the manufacturer's instructions. The primer sequences used to amplify the human H19 transcript was (5_-ACTGGAGACTAGGGAGGTCTCTAGCA) upstream and (5_-GCTGTGTGGGTCTGCTCTTTCAAGATG) downstream. The polymerase chain reaction (PCR) was carried out for 30 cycles (98°C for 15 sec, 58°C for 30 sec, and 72°C for 40 sec and finally 72°C for 5 min). The integrity of the cDNA was assayed by RT-PCR analysis using the histone variant, H3.3 or GAPDH as positive control. The products of the PCR reaction were run on 2% agarose in TAE electrophoresis running buffer (40 mM Tris acetate and 2 mM EDTA, pH 8.5), stained by ethidium bromide and visualized by UV.

### Human ascites fluid

The Ascitic fluid samples from the peritoneum of patients suffering from ovarian cancer were submitted to this study following approval of the Israeli Ethics Committee. Samples were kindly given to us from the Division of Gynecologic Oncology, Wolfson Medical Center, and from the Department of Gynecology, Hadassah Medical Center. Cells were isolated by using centrifugation of a 15%, 30% and 60% percoll gradient. Cells from the 30% and 60% percoll gradient were used for RNA isolation and ISH analyses. Cells from each isolation (whenever possible), were fixed by 4% PFA on poly-lysine slides and dehydrated, submitted to RNA extraction and seeded in a 750 ml flask.

### Immunohistochemistry (IHC)

Following fixation IHC was performed on the isolated ascites cells. The LEVEL1 PEROXIDASE ANTI-PEROXIDASE (PAP) DETECTION SYSTEM and the CA-125 monoclonal antibody (Signet Laboratories Inc. Dedham, MA) were used to detect the CA-125 levels in ascites cells according to manufacture procedure. The fixated cells were rehydrated in PBS*1 at room temperature and were separately incubated with two blocking reagents (hydrogen peroxidase and normal serum) to reduce nonspecific background staining. Cells were then sequentially incubated with three antibody preparations: 1) primary antibody- the CA-125 monoclonal antibody, 2) linking antibody used to bind the primary antibody, 3) labeling antibody, peroxidase labeled mouse immunoglobulin to mark the antigen location. After adding substrate solution cells were counterstained with Mayer's hematoxilin.

### Dig-labeled probe synthesis and in situ hybridization

Digoxigenin labeled H19 RNA transcripts were produced by labeling with DIG-11-UTP by SP6, T3, or T7 RNA polymerase in an *in vitro *transcription reaction (Boehringer, Mannheim, Germany) as described before [26]. The preparation of the sections for *in situ *hybridization was as described [16]. Finally, the sections were counterstained with 3% Giemsa stain, quickly dried, and mounted in Enthelan. Hybridization was conducted with a sense RNA probe as control to test the specificity of the ISH. The intensity of the hybridization signal was indicated as (+1) for weak, (+2) for moderate and (+3) for strong signals. The distribution of the hybridization signal was referred to as focal (20%–70% of the cells) and defused (>70% of the cells).

### Animal heterotopic model for In-vivo DNA based drug

CD-1 or athymic female nude mice (6–8 weeks old, 20–25 g) were used for all the experiments.

All of the surgical procedures and the care given to the animals were approved by the local committee for animal welfare. The histopathological examination of the different tumors was performed in consultation with a trained pathologist.

#### Heterotopic model

Confluent ES-2 human ovarian carcinoma cells were trypsinized to a single cell suspension and resuspended in 2*10^6 ^cells/100 μl PBS, then subcutaneously injected into the back of 6–8 weeks old CD-1 or athymic female nude mice. 10 days after cell inoculation, the developed tumors were measured in two dimensions and subjected to different treatments. Intratumoral injections of 25 μg of the toxin construct DTA-H19 and 25 mg of the reporter vector Luc-H19 (control group) were performed at days 10, 12, 14 and 16 after cell inoculation. Tumor dimensions were measured, and the tumor volume was calculated according to the formula (width)^2 ^× length × 0.5. The animals were sacrificed 3 days after the last injection, the tumors were excised and their *ex-vivo *weight and volume were measured.

## Results

### The level of H19 transcript in ascites from different patients detected by ISH or by RT-PCR

**Figure 1:**
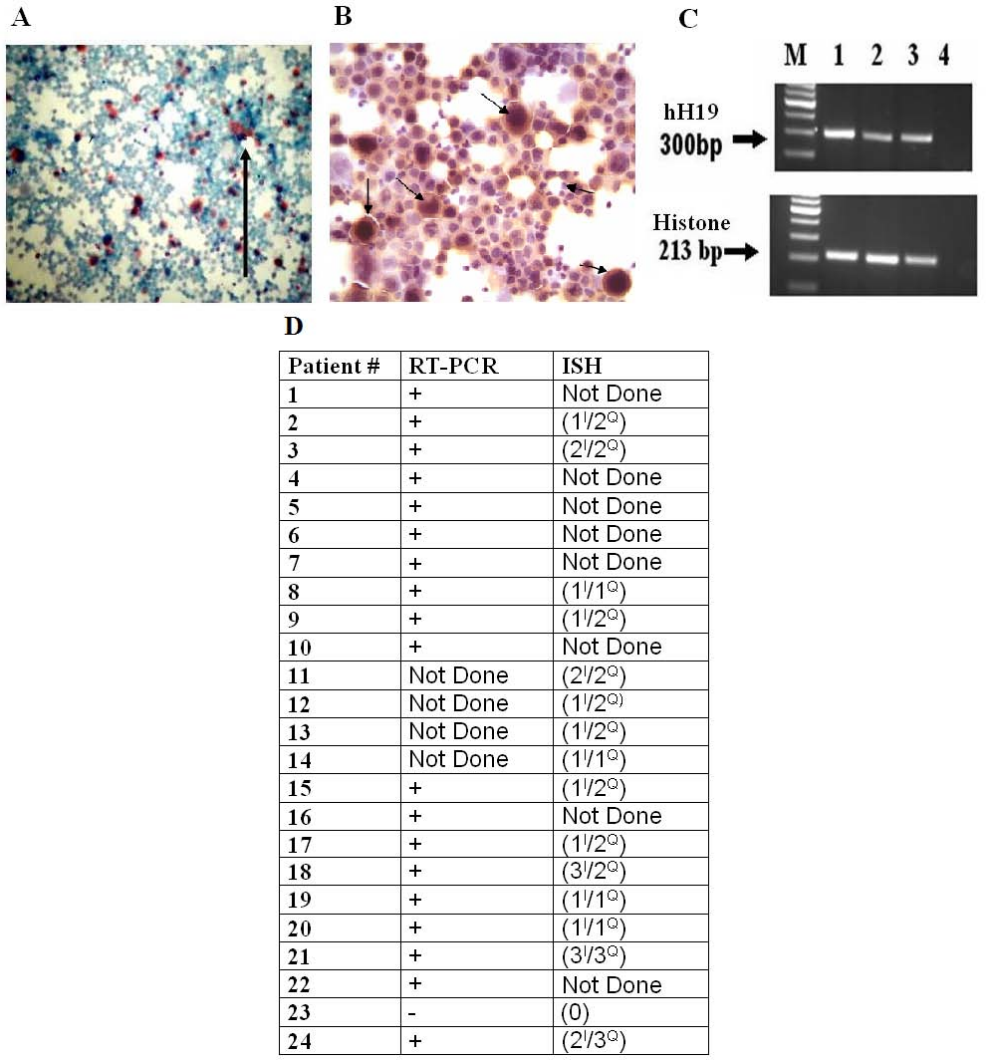
**The level of H19 transcript in RNA isolated from cells of ascites fluid of different patients determined by RT-PCR or by ISH**. A. H19 transcripts in the isolated ascites cells determined by ISH analysis. A positive stained cell is marked by a black arrow. B. The level of CA-125 in cells isolated from ascites fluid determined by IHC analysis (× 40 magnification). Black arrows mark the strong positive stains of cells expressing CA-125. C. The H19 transcript in RNA extracted from ascites cells determined by RT-PCR analysis. "M" 100-bp molecular weight marker. Line 1 – patient #1, Line 2 – patient # 2, Line 3 – patient # 3 and Line 4 – negative control. D. RT – PCR and ISH analysis of ascites cells from different patients. The RT-PCR results are expressed as positive (+) or negative (-). The ISH results are expressed as the number of moderate to strongly H19 positive samples. The intensity of hybridization signal was indicated as (+1) for weak, (+2) for moderate and (+3) for strong signals. The quantity of the staining was referred to (+1) up to one third of the cells, (+2) one to two thirds of the cells and (+3) more than two thirds of cells (I-indicates the intensity of the signal, Q-indicates the quantity of signal). Some samples could not be analyzed due to lack of material.

To evaluate the possible use of H19 regulatory sequences for the therapy of ovarian cancer, we determined the level of H19 transcripts in cells from ascites fluid of women patients. Ascitic fluid was collected from the peritoneum of patients carrying ovarian cancer. The ascites cells were separated from contaminating cells (mainly blood cells) on a percoll gradient. The isolated ascites cells were then either fixed on slides for ISH and immunohistochemistry (IHC) analysis (Figure 1A+B), in addition, total RNA was extracted and the level of H19 RNA was determined by RT-PCR analysis (Figure 1C).

We studied the H19 gene expression in 24 different women patients using RT-PCR and ISH techniques. Figure 1D shows the expression of H19 gene in ovarian cancer cells from ascites fluid from different human patients. A semi quantitative scoring system was established to define the levels of H19 expression after ISH (see "Material and Methods").

Figure 1(A+C) shows high levels of H19 transcripts in ascites cells collected from the patients. All patients tested were positive for H19 gene expression. Figure 1A also shows that when H19 transcripts were determined by ISH, a strong positive staining was detected in the cell's cytoplasm. To confirm that the isolated ascites cells originated from ovarian carcinoma, the level of ovarian cancer tumor marker, CA-125, was examined by IHC on some of the slides obtained from the patients (Figure 1B). Positive staining of CA-125 glycoprotein in the ascites cells is shown in Figure 1B which indicates that the cells isolated from the ascites fluid are ovarian cancer cells.

Figure 1D Figure 1D shows that in 23 cases out of 24, ascites cells were positive for H19 transcript (96%). 19 out of 20 (95%) ascites samples examined by RT-PCR showed H19 expression. The ISH analysis showed that in 15 out of 16 (93%) patients the H19 gene was expressed. High and moderate levels of the H19 transcript were detected in 12/15 (74%) samples (samples indicated as 1^I^/2^Q ^were considered as moderate levels of H19). Only 26% (4/15) of the samples tested showed low levels of H19 transcript (indicated as 1^I^/1^Q^).

Based on these results, we decided to further investigate the use of H19 regulatory sequences for driving toxin gene expression in a therapeutic vector for ovarian cancer.

### The level of H19 transcript in human ovarian cancer cell lines

**Figure 2:**
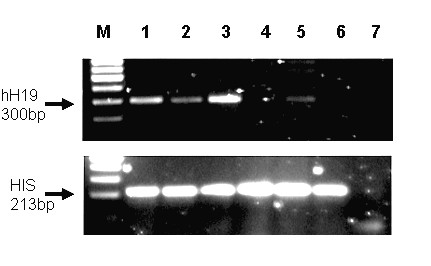
**The level of the H19 transcript in human ovarian cell lines determined by RT-PCR**. "M" 100-bp molecular weight marker. Line 1 – OVCAR-3, Line 2-SKOV-3, Line 3 – OV-90, Line 4 – CA-OV3, Line 5 – TOV-112D, Line 6 – ES-2 and Line 7 – negative control. The upper panel indicates the 300 bp H19 cDNA and the lower panel indicates the 300 bp histone internal control.

We determined the level of H19 RNA in different human ovarian cancer cell lines. Total RNA was extracted from the cell cultures. The levels of H19 transcripts were detected by RT-PCR analysis in the following cell lines: OVCAR-3, SKOV-3, OV-90, CA-OV3, TOV-112D and ES-2 are shown in Figure 2.

Figure 2 showed different levels of the H19 gene expression. H19 transcripts were detectable in OVCAR-3, SKOV-3, OV-90 and TOV-112D cell lines, while no detectable levels of the H19 transcript were noted in the CA-OV and ES-2 cell lines.

### The activity of the human H19 promoter cloned into the Luc-H19 plasmid and the killing effect of the DTA-H19 vector in ascites from patient #1 and in different cell lines

**Figure 3:**
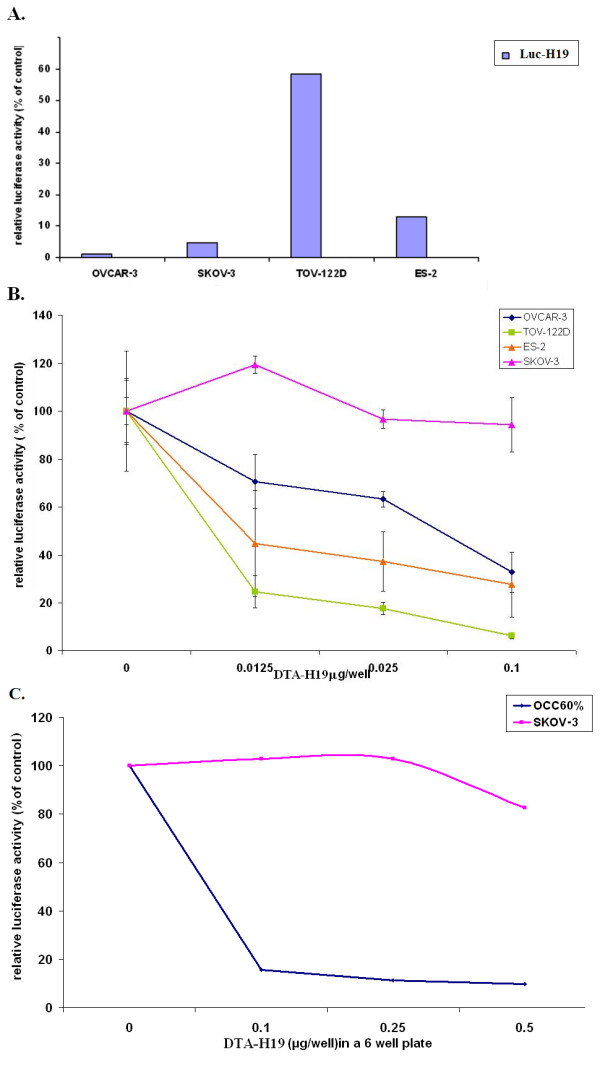
**Relative luciferase activity induced by transfection of human cell lines with Luc-H19 plasmid and the reduction of luciferase activity in human ovary primary culture from patient #1 and in human ovarian carcinoma cell lines due to co-transfection with the DTA-H19 vector**. A. Relative luciferase activity, in OV-CAR, SKOV-3, TOV-112D and ES-2 human cell lines induced by transfection with Luc -H19 plasmid. Each cell line was transfected with 2 μg of Luc -H19 or the LucSV40 plasmid. The values represent the luciferase activity of the H19 promoter relative to the activity of the control vector LucSV40. B. The killing potential of the DTA-H19 vector in OVCAR-3 (blue), SKOV-3 (pink), TOV-112D (green), and ES-2 (orange) was measured as a reduction of LucSV40 activity. Cells were cotransfected with 2 μg LucSV40, and the indicated concentrations of DTA-H19 or LucSV40 alone. C. The killing potential of the DTA-H19 vector in human primary culture (blue) compared with SKOV-3 (pink) was measured as a reduction of Luciferase activity. Cells were transfected with 3 μg of LucSV40 alone, or cotransfected with 3 μg LucSV40 and the indicated concentrations of DTA-H19. Transfection experiments were stopped after 48 hours and luciferase activity was assessed. The activity of the luciferase in the LucSV40 transfected cells was compared to the luciferase activity in the cotransfected cells.

The transcriptional activity of the H19 regulatory sequences cloned into the DTA-H19 plasmid was examined in a variety of cell lines. The luciferase activity induced by the H19 regulatory sequences (Luc -H19 plasmid) was determined in those human cell lines that were previously analyzed for endogenous H19 transcripts expression (Figure 2). Cells were transfected with 2 μg/well of the indicated vectors and luciferase activity was measured by luciferase assay (Figure 3A). Next we also tested the *in-vitro *killing potential of the DTA-H19 plasmid in the same human ovarian cancer cell lines. OVCAR-3, SKOV-3, TOV-112D and ES-2 cell lines were cotransfected with 2 μg of LucSV40 and the indicated concentrations of DTA-H19 (Figure 3B). Luciferase activity was determined and compared to that of cells transfected with LucSV40 alone. In addition, the killing potential of the DTA-H19 plasmid was tested in ascites from patient #1 (OCC 60%) and in DT-A resistant ovarian carcinoma cell line SKOV-3 as control. Cells were cotransfected with 3 μg of LucSV40 and the indicated concentrations of DTA-H19 (Figure 3C).

The relative reduction of the luciferase activity in the cotransfected cells reflect the level of the H19 driven DT-A expression and thus cell killing.

The results in Figure 3A showed the relative luciferase activity in the different cell lines which measured the H19 regulatory transcriptional activity in each cell line. Extremely high luciferase activity was detected in the TOV-112D cell line while relatively low levels were detected in OVCAR-3, SKOV-3 and ES-2 cell lines. The levels of H19 transcripts in different cell lines (Figure 2) were not always in accordance with the relative luciferase activity as shown in Figure 3A. This can be explained by the existence of additional regulatory sequences found in the endogenous H19 gene which were not cloned into the plasmid containing the human H19 regulatory sequences, or by differential stability of the H19 RNA in different cell lines.

A significant decrease in luciferase activity was detected in the cotransfected cell lines (Figure 3B), and in the cotransfected cells obtained from ascites of a patient with advanced ovarian cancer (Figure 3C). The relative reduction of the luciferase activity in the cotransfected cells is completely dependent on hH19 driven DT-A expression and thus cell killing. The H19 promoter is able to drive the expression of the DT-A gene and thus causing inhibition of protein synthesis and cell death which lead to the reduction of LucSV40 activity. The decrease in each cell line is in a dose-response manner. Reduction in luciferase activity of 30%, 75% and 55% (P < 0.003) was obtained after cotransfection of OVCAR-3, TOV-112D and ES-2 cells respectively, with 2 μg/well of LucSV40 and 0.0125 μg/well of the DT-A expressing plasmid (Figure 3B). On the other hand, the diphtheria toxin resistant cell line, SKOV-3 showed no or very low decrease in luciferase activity (Figure 3B and 3C). Moreover, a positive correlation between luciferase activity induced by H19 regulatory sequences shown in Figure 3A and the reduction in luciferase activity due to DTA expression (Figure 3B) can be noted.

In the cotransfected experiments, as the amount of LucSV40 is much larger than those of DTA-H19 plasmid, one can assume that the decrease of luciferase activity is not due to a competition for cell penetration with the DTA-H19 construct, causing a reduction in the amount of LucSV40 which entered the cells, but is a direct consequence of the H19 promoter driven expression of DT-A.

These results justify the use of a DNA based drug in which a toxin is produced under the control of H19 regulatory sequences.

### The level of H19 transcripts in heterotopic subcutaneous tumors

**Figure 4:**
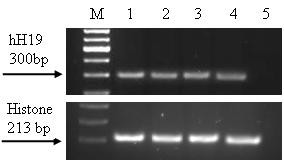
**The level of H19 transcripts in heterotopic subcutaneous tumors after injection of the ES-2 cells determined by RT-PCR**. "M"100-bp molecular weight marker. Lines 1–4 – heterotopic subcutaneous tumors from different mice and Line 5 – negative control. The sizes of the PCR products are 300 bp and 213 bp for human H19 and Histone internal control respectively.

In order to develop a model for heterotopic ovarian tumors, ES-2 ovarian carcinoma cells were subcutaneously injected into the dorsa of 6–7 week old CD-1 female mice. Tumors were developed after 9 days and were dissected 14 days after cell injection. Total RNA was extracted from the frozen tumors. The level of H19 RNA was determined by RT-PCR analysis (Figure 4).

Although no H19 expression was detected in the ES-2 cell line, (Figure 2, line 6), significant H19 RNA was detected in all the developed tumors examined (Figure 4), supporting the role of H19 in tumor growth [12]. Thus, the results shown in Figure 4 indicated that the use of this cell line is suitable to establish an ovarian carcinoma animal model. Moreover, the ES-2 cell line causes rapid development of the tumor.

### In-vivo tumor growth inhibition by DTA-H19 vector

We used the DTA-H19 vector for evaluating its therapeutic potential by DT-A expression *in-vivo *using the animal models for ovarian cancer.

### Treatment of heterotopic subcutaneous tumors

**Figure 5:**
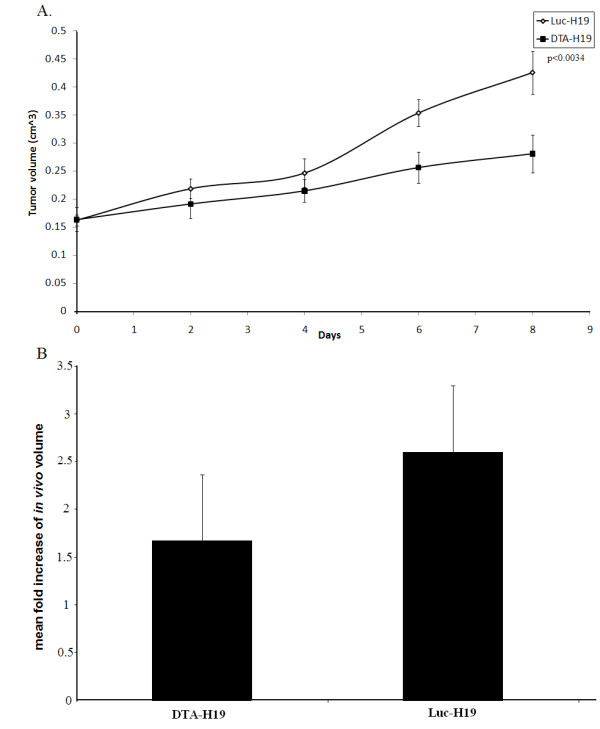
**The effect of direct intratumoral injection of the DTA-H19 plasmid on subcutaneous ovarian tumor growth in nude mice**. 24 mice were injected with the ES-2 cells. Starting on day 10, 12 mice received 4 injections of 25 μg of DTA-H19 plasmid and the other 12 mice received 4 injections of 25 μg of Luc-H19 plasmid complexed with PEI. Injections were given with two-day intervals. One day after the last treatment, animals were sacrificed. The tumor dimensions were measured *in situ *prior to the treatment with the plasmid and after sacrifice. The effect of treatments with DTA-H19 or Luc-H19 plasmids on tumor volumes (cm^3^) over time (days) is indicated (A), while day 0 represents the first treatment given. The mean fold increase of the final volume was compared to the initial volume in the DTA-H19 and Luc-H19 treated tumors (B).

The ability of the DTA-H19 to promote cancer cell killing and inhibit tumor growth *in-vivo *was analyzed. ES-2 cells were subcutaneously injected into the back of 6–7 weeks old athymic female mice in order to develop a model for heterotopic ovarian cancer. 10 days after the subcutaneous cell inoculation, the mice developed measurable heterotopic tumors. Mice were randomly divided into two groups: A DTA-H19 group of 12 mice were intratumoral injected with 25 μg of the DTA-H19 plasmid and another group of 12 mice were intratumoral injected with 25 μg of the control plasmid Luc-H19. Both plasmids were injected as complexed with the transfection reagent jetPEI™ (DTA-H19/PEI and Luc-H19/PEI respectively). The sizes of the tumors were determined before each treatment (Figure 5A), and *in-vivo *fold increase of the tumor size was calculated (Figure 5B).

Figure 5A shows that while similar tumor volumes in the two groups of mice were measured on day 0 (day of the first treatment), inhibition in the rate of tumor growth was detected after each treatment with DTA-H19/PEI plasmid as compared to the tumor growth of Luc-H19/PEI treated mice (p < 0.034). In addition, Figure 5B shows that 4 injections of DTA-H19/PEI plasmid in two-day intervals were able to inhibit tumor growth by 40% compared to 4 Luc-H19/PEI treatments (P < 0.05).

## Discussion

The present work shows the use of the regulatory sequences of the H19 gene for the development of DNA-based therapy for human ovarian cancer related ascites. The successful development of anti-tumor gene therapy depends on the use of a combinatorial approach aimed at targeted delivery and specific expression of effective anti-tumor agents. Various gene therapy strategies for the treatment of ovarian cancer are currently under development and aim towards maximal treatment efficacy and minimal adverse effects. In this study, a tumor-selective promoter was used in conjunction with a cytotoxic gene to achieve targeted tumor cell destruction. Trials in animal models showed that tumor specific promoters exhibit a clear advantage compared to strong viral promoters such as CMV promoter currently used in clinical trials [27]. While most tumor-specific promoters are relatively weak, resulting in insufficient transgene expression levels, the H19 promoter is known to be highly activated in various tumor types and to show no or only marginal activity in the surrounding normal tissue [26,28].

The goal of the present study was to evaluate the therapeutic potential of expression vectors carrying the "A" fragment of the diphtheria toxin (DT-A) gene under the control of the H19 regulatory sequences in an ovarian carcinoma animal model. We have previously shown that these constructs are able to selectively kill tumor cell lines and inhibit tumor growth *in vitro *and *in vivo *[[28,24,23] and [22]]. The choice of the DT-A as a toxin gene ensured not only high killing activity but its use has a great advantage in avoiding unintended toxicity to normal cells, since the DT-A protein released from the lysed cells is not able to enter neighboring cells in the absence of the DT-B fragment [29].

In order to determine the feasibility of this approach for the therapy of ovarian cancer in a human patient, both RT-PCR and ISH analyses were applied on cells isolated from OCAF to determine the level of H19 gene expression. High levels of H19 transcript were detected in the ascites malignant cells (Figure 1A+B+C). The high level of H19 RNA found in the OCAF is in accordance with previous results obtained from our study on the expression profile of H19 in epithelial ovarian cancer [19].

The therapeutic potential of the toxin vector was evaluated *in vitro *using different human ovarian cancer cell lines and in cells isolated from OCAF (Figures 3A and 1B). The H19 regulatory sequences were able to drive DTA expression in the ovarian cancer cell lines that led to cell death. Therefore, we further investigated the therapeutic potential of the toxin vector *in vivo *using the ES-2 cell line which has high tumorogenic properties. Although ES-2 cells (carrying a p53 mutation) showed no endogenous expression of the H19 gene when tested in culture (Figure 2, lane 6), H19 RNA were detected at high levels in all the tumors developed following injection of these cells into the animal (Figure 4), supporting the possible role of H19 in tumor growth which is upregulated under hypoxic stress. In addition, it was previously shown that in certain bladder carcinoma cell lines H19 RNA is either not or weakly expressed in normal culture conditions, but strongly expressed when tumors are grown by injecting these cell lines into nude mice [30,31].

H19 expression was also detected in ascites developed after intraperitoneal injection of these cells into the peritoneum of nude mice (data not shown). Furthermore, the apparent non-correlation between transgene expression under the regulation of the H19 promoter and endogenous H19 expression might be explained by the absence of negative regulatory sequences in the DTA-H19 construct. The promoter activity of endogenous H19 gene is determined by the naked chromatin structure which differs from that of the constructs transfected into the cells. Thus, transcription factors may be able to induce transcription from the plasmid, but not from the endogenous gene.

We have shown the existence of a tight association between the p53 status and H19 induction under hypoxic stress (manuscript sent for publication). In this case, it is possible that the enhanced H19 expression observed in these tumors is related to selection and clonal expansion of H19 expressing cells, under the severe and harsh conditions (for example: low oxygen levels) of a rapidly growing tumor in vivo, which is the real situation in the target tumors to be treated.

The heterotopic model for ovarian cancer used in this research has the advantage of rapidly developing tumors, allowing short turn-around times for the experiments (three weeks). In addition, the developed tumors are easily manageable because of relatively large size and accessibility. The DTA-H19/PEI complex was able to highly inhibit the growth rate of the subcutaneous tumors induced in mice by subcutaneous injection with the ES-2 cell line (Figure 5A). At least 40% inhibition of tumor growth by DTA-H19/PEI was obtained compared to tumors treated with the control plasmid Luc-H19/PEI (P < 0.05) (Figure 5B). Moreover, it is very important to note that no signs of unwanted toxicity were detected in normal mice treated subcutaneously by DTA-H19/PEI.

We used the cationic polymer PEI (JetPEI™), a linear polyethylenimine derivative as a transfection promoter agent in the heterotopic animal model described in Figure 5. The JetPEI™ compacts the DNA into positively charged particles capable of interacting with anionic proteoglycans at the cell surface, thereby facilitating the entering of the DNA by endocytocis [32]. No toxic effect was detected in the treated animals participating in these experiments.

Although this is a preliminary study, our working hypothesis is that intraperitoneal administration of DTA-H19 has the potential to reach ascites tumor cells, deliver its intracellular toxin without targeting normal tissues, and thus may help reduce tumor burden, fluid accumulation; improve the quality of life of the patient; and prolong their life span. This suggested approach was further demonstrated in a compassionate patient treatment in which the DTA-H19 plasmid was intraperitoneally injected into the peritoneum of a woman with advanced and recurrent ovarian carcinoma. Following several infusions, a complete resolution of ascites was shown (Case report in preparation).

## Conclusion

On the basis of this study we formed a platform for the design of an extensive phase I study on a larger number of human patients to test the safety of this treatment.

The results obtained in the present study may represent the first step in a major breakthrough in the treatment of human OCAF. Data regarding the correlation between the level of H19 expression and the efficacy of the treatment should be collected during a Phase I and II clinical trials which are being planned. Based on the data collected during these future clinical trials we will be able to identify responders from non-responders in advance who are resistant to all known therapies, thereby avoiding treatment failure coupled with unnecessary suffering and cost.

## Abbreviations

ATCC: American type culture collection; CA-125: Cancer Antigen 125; DT-A: diphtheria toxin A chain; DTA-H19: vector expressing the DT-A gene under the control of H19 regulatory sequences; EOC: Epithelial ovarian cancer; IHC: Immunohistochemistry; ISH: In situ hybridization; Luc: luciferase gene; Luc-H19; reporter vector expressing the luciferase gene under the control of H19 regulatory sequences; Luc4/LucSV40: reporter vector expressing the luciferase gene under the control of SV40 promoter and enhancer; OCAF: Ovarian cancer ascites fluid; PCR: polymerase chain reaction; PEI: polyethylenimine; SV40: simian virus 40; TCC: transitional cell carcinoma.

## Competing interests

The authors declare that they have no competing interests.

## Authors' contributions

AM conducted the study, participated in design, coordination, data interpretation, performed the statistical analysis, and drafted the manuscript. AC participated in the study design and coordination. TL participated in the analyses of the ovarian ascites fluid. SA participated in the *in vitro *studies. JG participated in the *in vitro *studies. IM participated in the *in vivo *studies. RA participated in the PCR studies. VS participated in the *in vivo *studies. TB participated in the *in vivo *studies histology, IHC, ISH interpretation. NDG helped to draft the manuscript and data interpretation. AH conceived of the study, participated in design, interpretation of data, and critically revised the manuscript. PO participated in design, coordination, and data interpretation and drafted the manuscript. All authors read and approved the final manuscript.
